# Development of Human Serum Albumin Selective Fluorescent Probe Using Thieno[3,2-*b*]pyridine-5(*4H*)-one Fluorophore Derivatives

**DOI:** 10.3390/s19235298

**Published:** 2019-12-01

**Authors:** Suji Lee, Dan-Bi Sung, Seungyoon Kang, Saravanan Parameswaran, Jun-Ho Choi, Jong Seok Lee, Min Su Han

**Affiliations:** 1 Department of Chemistry, Gwangju Institute of Science and Technology (GIST), 123 Cheomdangwagi-ro, Buk-gu, Gwangju 61005, Korea; 2 Marine Natural Products Chemistry Laboratory, Korea Institute of Ocean Science and Technology (KIOST), Busan 49111, Korea; 3 Department of Marine Biotechnology, Korea University of Science and Technology, Daejeon 34113, Korea

**Keywords:** human serum albumin, fluorescent probe, thieno[3,2-*b*]pyridine-5(*4H*)-one, microalbuminuria, intramolecular charge transfer

## Abstract

The level of human serum albumin (HSA) in biological fluids is a key health indicator and its quantitative determination has great clinical importance. In this study, we developed a selective and sensitive fluorescent HSA probe by fluorescence-based high-throughput screening of a set of fluorescent thieno[3,2-*b*]pyridine-5(*4H*)-one derivatives against major plasma proteins: HSA, bovine serum albumin (BSA), globulin, fibrinogen, and transferrin. The fluorophore chosen finally (**4**) showed noticeable fluorescence enhancement in the presence of HSA (160-fold increase), and it exhibited rapid response, high sensitivity (detection limit 8 nM), and the ability to clearly distinguish HSA from BSA in pH 9 buffer condition. Moreover, the probe could be applicable to detect trace amounts of HSA in an artificial urine sample; further, it might be applied to the determination of the HSA concentration in complex biological samples for pre-clinical diagnosis.

## 1. Introduction

Human serum albumin (HSA) is the most abundant protein in blood plasma; generally, its concentration is maintained at levels 35–55 g/L in normal serum and 30 mg/L in normal urine. HSA has multiple functions in the human body: it plays a major role in maintaining the oncotic pressure and in the transport of various drugs and metabolites [1,2,3,4,5,6]. Thus, an abnormal HSA level in a body fluid can be closely associated with many health problems. Hypoalbuminemia, defined as a very low HSA concentration in serum, is frequently observed in patients with malnutrition and cirrhosis [7,8]. In addition, microalbuminuria (high level of HSA in urine) is related to kidney disease in diabetes mellitus and hypertension [9,10]. HSA concentration is a key health-related indicator and its quantitation in biological fluids is of great significance for diagnosis [11].

To date, various HSA detection methods have been developed, such as immunoassay, LC-MS/MS-based proteomics, capillary electrophoresis, colorimetric, and fluorescent probes [12,13,14,15,16,17,18,19,20,21]. In comparison with other detection methods, fluorescent probes are favored because of advantages including a rapid, non-invasive, and sensitive response. Accordingly, several fluorescent probes have been reported using many different types of fluorophore such as tetraphenylethylene, squaraine dye, and indolium derivative, which is based on aggregation-induced emission (AIE), molecular rotor containing donor-π-acceptor (D-π-A) structured fluorophore, and self-assembled nanoparticles [22,23,24,25,26,27,28,29,30,31,32,33,34,35,36,37]. However, there remain some problems to be solved, such as the low sensitivity and selectivity toward HSA. They could successfully detect high levels of HSA in serum but were not applicable to detect trace amounts of HSA in urine. Furthermore, most could hardly distinguish HSA from bovine serum albumin (BSA), which has a similar structure but different functional level.

**Scheme 1:**
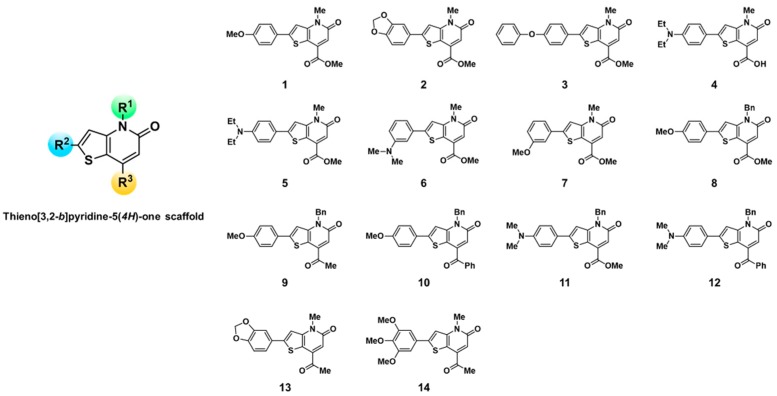
Structures of a set of KIOST-Fluor (KF) derivatives, **1**–**14**, used to develop a human serum albumin (HSA)-selective fluorescent probe.

To develop a novel fluorescent probe having high sensitivity and selectivity for HSA, we utilized the fluorescence-based, high-throughput screening of a set of novel fluorophore derivatives against plasma proteins (Scheme 1). Recently, we have reported novel fluorophore scaffolds: thieno[3,2-*b*]pyridine-5(*4H*)-one derivatives [hereafter, collectively referred to as KIOST-Fluor (KF)], synthesized via a series of reactions including the Suzuki-Miyaura cross-coupling reaction and a regioselective aza-[3+3] cycloaddition of 3-aminothiophenes with α,β-unsaturated carboxylic acids [38]. By varying the functional group on the KF scaffold, the fluorophore showed different photophysical properties; in particular, they exhibited remarkable environmental sensitivities. 

Herein, we report a new fluorescent probe for the highly selective and sensitive detection of HSA using the environment-sensitive properties of the KF system. We have demonstrated the HSA-sensing performance of this probe in terms of sensitivity, selectivity, binding properties, and applicability; this fluorescent probe exhibited superior selectivity over BSA with a 160-fold fluorescence intensity increase. In addition, we explored the application of this fluorescent probe to the detection of trace amounts of HSA spiked in artificial urine. A rapid-response and highly sensitive probe for HSA using this novel fluorophore scaffold would greatly contribute to clinical diagnosis.

## 2. Materials and Methods

### 2.1. Materials and Instruments

All the biological analytes, HSA, BSA, γ-globulin from human blood, fibrinogen from human plasma, transferrin from human, hemoglobin from human, haptoglobin from human, chymotrypsin from bovine pancreas, trypsin from bovine pancreas, lysozyme from pooled human plasma, and butyrylcholinesterase (BChE) from equine serum were purchased from Sigma-Aldrich (St. Louis, MO, USA) and used without further purification. High-throughput screening results were recorded by a Cytation 3 Multi-Mode Reader (BioTek, Winooski, VT, USA) using a 96-well plate. UV-vis spectra were recorded by a JASCO V-630 Spectrophotometer (JASCO Inc., Easton, MD, USA), and fluorescence spectra were recorded by a Cary Eclipse Fluorescence Spectrophotometer (Agilent, Santa Clara, CA, USA) using a quartz cell of path length 1 cm.

### 2.2. Synthesis of KF Derivatives (Thieno[3,2-b]pyridine-5(4H)-one Derivatives)

General synthetic procedure for KF derivatives can be found in our previous report [38]. The detailed synthetic procedures of KF **1**–**12** were previously reported and that of KF **13** and KF **14** were described in Supplementary Material.

### 2.3. Fluorescence-Based High-Throughput Screening of KF Derivatives against Major Plasma Proteins

Stock solutions of all KFs, **1**–**14**, (50 μM) were prepared in dimethyl sulfoxide (DMSO). Samples containing each fluorophore (5 μM, 10% DMSO) under different buffer conditions (pH 5 acetate, pH 7 Tris, pH 9 Tris; 20 mM) were prepared in 96-well plates and the absorption wavelengths of the fluorophores were recorded by the Cytation 3 Multi-Mode Reader.

Stock solutions of five major plasma proteins (100 μM) including HSA, BSA, γ-globulin, fibrinogen, and transferrin were prepared in distilled water. Fluorophores-containing samples (5 μM in 10% DMSO), with and without proteins (10 μM), were prepared under various buffer conditions (pH 5 acetate, pH 7 Tris, pH 9 Tris; 20 mM) in a 96-well plate; fluorescence spectra were recorded by the Cytation 3 Multi-Mode Reader at different excitation wavelengths (λ_ex_) (370, 400, 420, and 450 nm).

### 2.4. Comparison of Fluorophores as Human Serum Albumin (HSA) Probe Candidates in Terms of Selectivity

Stock solutions of KF **4** and **11** were prepared in DMSO, and stock solutions of HSA and BSA were prepared in distilled water. Firstly, UV-vis spectra of the samples containing each KF **4** and **11**, (5 μM in 10% DMSO) with and without HSA (1, 5, 10, 20, 50, and 100 μM) or BSA (50, 100 μM) were recorded in pH 9 buffer (Tris, 20 mM) using an UV-vis spectrophotometer. Then, blanks, each containing only one fluorophore (5 μM), and samples, containing each fluorophore (5 μM, 10% DMSO) with different concentrations of albumin (HSA or BSA, 5, 10, and 20 μM), were prepared in a pH 9 buffer solution (Tris, 20 mM). The fluorescence spectra were recorded by the fluorescence spectrophotometer under excitation at 455 nm for **4** and at 440 nm for **11**.

### 2.5. The Effects of pH and DMSO on the HSA-Sensing Behavior of ***4***

To investigate the effect of pH on the HSA-sensing performance using **4**, the fluorescence intensity at 546 nm of samples containing fluorophore **4** (5 μM, 10% DMSO) in absence and presence of albumin (HSA or BSA, 10 μM), in different pH buffers (pH 5, 6 acetate, pH 7, 7.4, 8, 9 Tris; 20 mM), were recorded by the fluorescence spectrophotometer under excitation at 455 nm. To investigate the effect of DMSO on the HSA-sensing performance using **4**, the fluorescence intensity at 546 nm of samples containing fluorophore **4** (5 μM) in absence and presence of albumin (HSA or BSA, 10 μM) with different ratios of DMSO (1, 5, 10%) were recorded under excitation at 455 nm. The experiment was repeated three times.

### 2.6. Determination of the Limit of Detection (LOD)

The fluorescence spectra of **4** (5 μM, 10% DMSO) with various concentrations of HSA (0, 0.05, 0.1, 0.3, 0.5, 0.8, 1, 3, 5, 10, 20, and 50 μM), in pH 9 buffer (Tris, 20 mM), were recorded by the fluorescence spectrophotometer under excitation at 455 nm; the titration experiment was repeated three times. The LOD was calculated by using the 3σ/slope rule based on the titration experiments, in which σ is the standard deviation of the blank measurements.

### 2.7. Determination of Quantum Yield of ***4***

To determine the quantum yield (Φ) of **4** in absence or presence of HSA, fluorescein in 0.1 M NaOH (Φ_ref_ = 0.95) was used as a reference. The quantum yield was calculated according to the following equation:

$$\phi =\phi_{ref}\left(\frac{m}{m_{ref}}\right)\left(\frac{n^{2}}{n_{ref^{2}}}\right),$$

where *m* is the slope of the line obtained from the plot of the integrated fluorescence intensity versus absorbance and *n* is the refractive index of solvent [27].

### 2.8. Selectivity Test of ***4*** toward HSA over Plasma Proteins

The fluorescence intensity at 546 nm of a blank containing only **4** (5 μM, 10% DMSO), in pH 9 buffer (Tris, 20 mM), and of samples containing **4** with each plasma protein (HSA, BSA 200 mg/L; γ-globulin, fibrinogen, and transferrin 50 mg/L; hemoglobin, haptoglobin, chymotrypsin, trypsin, and lysozyme 10 mg/L; and BChE 10 U/L) were recorded by the fluorescence spectrophotometer under excitation at 455 nm. The experiment was repeated three times.

### 2.9. Job’s Plot Analysis

Samples were prepared by mixing **4** with HSA at different ratios in pH 9 buffer (Tris, 20 mM), while maintaining the overall concentration ([4] + [HSA]) at 10 μM. Then, the fluorescence intensities of the samples at 546 nm were recorded by the fluorescence spectrophotometer under excitation at 455 nm. The experiment was repeated three times [32,39].

### 2.10. Determination of the Dissociation Constant (K_d_) of HSA and ***4***

The dissociation constant was calculated based on the method presented in previous literature reports based on the HSA titration results using **4** (5 μM, 10% DMSO) in pH 9 buffer (Tris, 20 mM) [27].

### 2.11. Assignment of the Binding Site of ***4*** on HSA

A stock solution of **4** in DMSO (100 μM) was prepared as well as those of site-specific binding drugs (Ibuprofen, Digitoxin, Warfarin, salicylic acid, 5 mM) in DMSO. Mixtures of HSA (10 μM) with each drug (500 μM) in pH 9 buffer (Tris, 20 mM) were incubated for 3 min, then **4** (5 μM, 10% DMSO) was added to the mixture and the fluorescence intensities at 546 nm were recorded by the fluorescence spectrophotometer under excitation at 455 nm. The experiment was repeated three times.

### 2.12. Identification of the Sensing Mechanism of ***4*** toward HSA

Using the Gaussian 09 program, calculations were performed to investigate the electronic transition of **4**. The identification of molecular orbitals (MOs) was accomplished by optimizing the geometry of **4** via a density functional theory (DFT) method, using the B3LYP hybrid functional and the 6-311G(d) basis set in the conductor-like polarizable continuum model (CPCM) solvent model. To demonstrate the viscosity-dependence of the fluorescence enhancement of **4**, the fluorescence intensity of **4** at 546 nm upon increasing the fraction of glycerol (0%, 10%, 20%, 30%, 40%, 50%, 60%, 70%, and 80%), in pH 9 buffer (Tris, 20 mM), were recorded by the fluorescence spectrophotometer under excitation at 455 nm.

Molecular docking is the widely adopted computational technique to identify the binding and intermolecular interactions between small molecules and biological macromolecules. The three dimensional structure of human and bovine serum albumin was downloaded from Protein DataBank database from RCSB website (PDB Id: 2BXG and 6QS9, respectively). The protein structures and probe **4** was preprocessed using AutoDockTools1.7.6 package with standard protocol prior to docking in AutoDock4.2 [40,41]. The docking was carried out with only polar hydrogens and Gasteiger charges. The grid box was constructed (90 × 90 × 90 points with grid spacing of 0.375 Å) to accommodate both primary and secondary binding site of human serum albumin because we expected that probe **4** would bind at Ibuprofen binding site. Molecular docking was carried out with lamarkian genetic algorithm for 100 runs with initial population size of 300 and number of evaluations of 1,000,000. The docking results were visually inspected with the help of Discovery Studio Visualizer [42].

### 2.13. Quantitative Detection of HSA in Artificial Urine

Artificial urine was prepared according to previous literature reports [43], and HSA stock solutions of various concentrations (5, 10, 25, 50, 80, 100, and 200 mg/L) were prepared both in artificial urine and in distilled water. To quantify the amount of HSA, the samples were prepared by mixing **4** (5 μM, 10% DMSO) with spiked HSA (20% artificial urine) in pH 9 buffer (Tris, 20 mM), and the fluorescence intensities of the samples were recorded by the fluorescence spectrophotometer under excitation at 455 nm. Then, the results were compared with those obtained in distilled water. The experiment was repeated three times.

## 3. Results and Discussion

### 3.1. Fluorescence-Based High-Throughput Screening of KF Derivatives against Major Plasma Proteins

To find a fluorophore having a high selectivity toward HSA, we screened fourteen KF derivatives against major plasma proteins (HSA, BSA, γ-globulin, fibrinogen, and transferrin) using a microplate reader. For a primary screening, we explored the absorption wavelengths (λ_abs_) of the fluorophore under different pH conditions, from acidic to basic (pH 5, 7, and 9), considering the pH-dependency of the KF derivatives. As shown in Table S1, the fluorophores had intrinsic absorption wavelengths ranging from 370 to 450 nm. We determined four characteristic excitation wavelengths (370, 400, 420, and 450 nm). Then, the fluorescence response of the KF derivatives in presence of each plasma protein were recorded at three different buffer conditions (pH 5, 7, and 9). Most of the KFs exhibited weak emission in absence of proteins, whereas several fluorophores (**2**, **4**, **5**, **9**, **10**, **11**, and **13**) showed significant fluorescence increases with two albumin equivalents (see Supplementary Material). We then selected as HSA probe candidates two fluorophores, **4** and **11**, which showed high selectivity toward HSA over BSA at basic conditions and remarkable fluorescence enhancement upon binding to albumin.

### 3.2. Comparison of Two Candidates in Absorption and Fluorescence Response toward HSA

**Figure 1:**
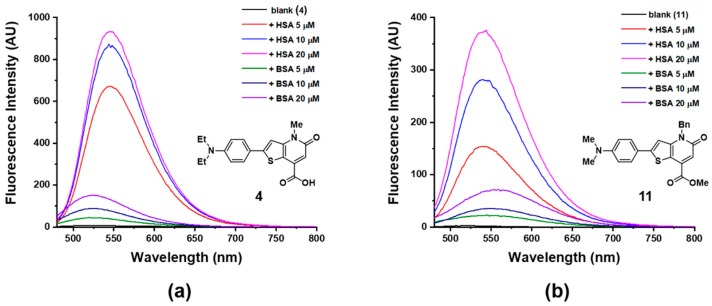
Fluorescence spectra of two fluorophores in absence and presence of human serum albumin (HSA) or bovine serum albumin (BSA), (**a**) Fluorophore **4** (5 μM in pH 9 Tris 20 mM, 10% DMSO), λ_ex_ = 455 nm; (**b**) Fluorophore **11** (5 μM in pH 9 Tris 20 mM, 10% DMSO), λ_ex_ = 440 nm.

Afterwards, we determined the most suitable fluorophore by comparing the two candidates **4** and **11** in terms of selectivity for HSA detection using a UV-vis- and a fluorescence spectroscopy. In the UV-vis spectroscopy study, we observed that the absorbance maxima of **4** and **11** gradually red-shifted with the addition of HSA (Figures S19 and S20). However, BSA addition had effect only on **11**; there was no change on the UV-vis spectrum of **4** even with a large amount of BSA. In relation to the fluorescence changes, **4** showed a 102-fold increase of fluorescence intensity with 1 equivalent of HSA (5 μM), while **11** showed only a 79-fold increase with an equal amount of HSA, as seen in Figure 1. In addition, **4** showed higher selectivity toward HSA over BSA than **11**, at even higher concentrations. Thus, **4** was chosen as the potential probe for HSA. To achieve a superior selective HSA-sensing performance using fluorophore **4**, the pH- and solvent-dependences of the HSA-from-BSA discrimination were investigated and the measurement conditions were optimized to pH 9 (Tris, 20 mM) containing 10% of DMSO (Figure S21).

### 3.3. Sensitivity and Selectivity of Probe ***4*** to HSA

**Figure 2:**
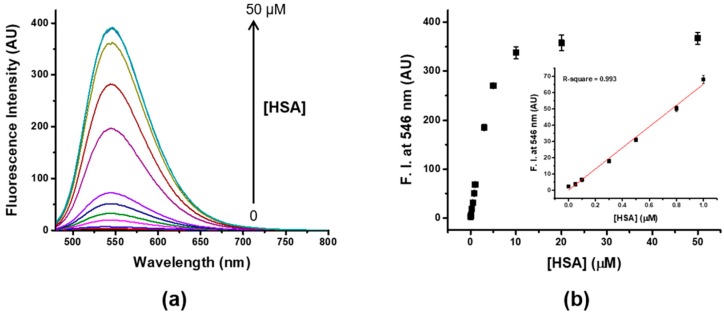
(**a**) Fluorescence spectra of **4** (5 μM, 10% DMSO) upon addition of increasing concentrations of HSA (0–50 μM) in pH 9 buffer (Tris, 20 mM), (**b**) Change of fluorescence intensity of **4** at 546 nm versus various concentrations of HSA; linear correlation of the fluorescence intensity with the concentration of HSA in the of 0–1 μM range (inset), λ_ex_ = 455 nm.

We examined the HSA-sensing behavior of fluorophore **4** under the optimized measurement conditions (pH 9.0 at 25 °C with 10% DMSO). As shown in Figure 2, fluorophore **4** showed a very weak emission at 526 nm in absence of HSA (Φ = 0.025), whereas the binding of **4** on HSA induced a slight red-shift of the emission band (from 526 to 546 nm) and an approximately 160-fold increase in fluorescence intensity at 546 nm with 2 HSA equivalents (Φ = 0.381). The fluorescence intensity of **4** increased linearly with the HSA concentration from 50 nM to 5 μM. The detection limit (3σ/slope) was determined to be 8 nM (532 μg/L), which is much lower than those of other reported probes. It is also much lower than the concentration of HSA in urine (30 mg/L), so it would be suitable to be applied in the quantitation of trace amounts of HSA in real biological fluids, including urine.

**Figure 3:**
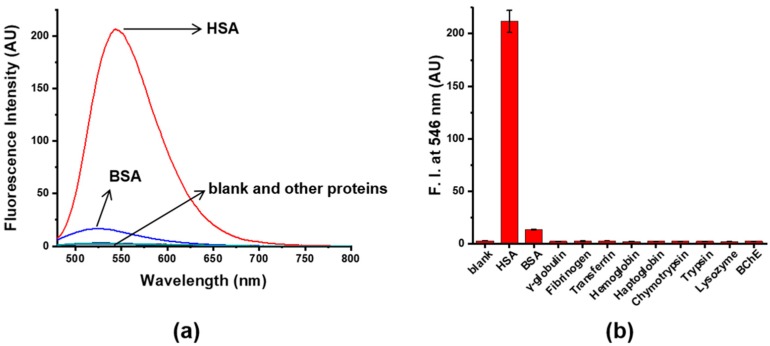
(**a**) Fluorescence spectra of **4** (5 μM, 10% DMSO) in absence and presence of a variety of plasma proteins including HSA (200 mg/L), BSA (200 mg/L), γ-globulin (50 mg/L), fibrinogen (50 mg/L), transferrin (50 mg/L), hemoglobin (10 mg/L), haptoglobin (10 mg/L), chymotrypsin (10 mg/L), trypsin (10 mg/L), lysozyme (10 mg/L), and BChE (10 U/L), in pH 9 buffer (Tris, 20 mM), (**b**) Fluorescence intensities, at 546 nm, of blank (**4** only) and sample containing **4** with plasma proteins, λ_ex_ = 455 nm.

Next, the selectivity of **4** toward HSA was investigated using diverse plasma proteins as well as BSA. As expected from the primary screening, other proteins including globulin, fibrinogen, transferrin, hemoglobin, haptoglobin, chymotrypsin, trypsin, lysozyme, and BChE induced negligible changes in fluorescence intensity, as seen in Figure 3. In addition, the discrimination of HSA and BSA was possible based on the relatively large fluorescence-response difference of **4** toward HSA and BSA, in emission wavelength and intensity, as shown in Figure 3a. In the presence of BSA, **4** showed an emission maximum at 526 nm and a slight increase in fluorescence intensity, while in the presence of HSA, **4** showed the emission maximum at 546 nm, slightly red-shifted from 526 nm, and its fluorescence intensity was 16-times higher than that in BSA. These results demonstrated, by the significant differences in emission properties, that probe **4** has superior selectivity toward HSA and can distinguish between HSA and BSA.

### 3.4. Binding Properties of Probe ***4*** on HSA

**Figure 4:**
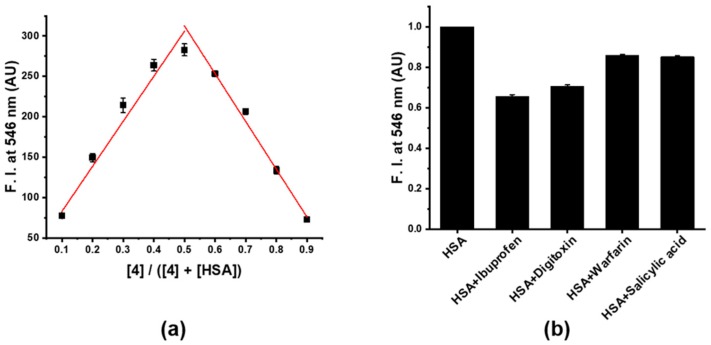
(**a**) Job’s plot analysis by measurement of the fluorescence intensity of a mixture containing **4** with HSA at different molar ratios, (**b**) Displacement of **4** from the HSA-**4** complex by site-specific binding drugs (500 μM).

A Job’s plot analysis was performed for the determination of the stoichiometry of the complex formed between **4** and HSA. As shown in Figure 4a, the maximum fluorescence intensity was observed at a 1:1 mole ratio of **4** to HSA, indicating one-site binding of **4** on HSA. In a further study, the measured dissociation constant (*K_d_*) was 5.4 μM, when a one-site binding model was fitted; this result showed that **4** could tightly bind to one-site of HSA with a relatively high affinity (Figure S22). Additionally, the measured *K_d_* of **4** and native HSA (*K_d_* = 5.4 μM) was not much different from that of delipidated HSA (*K_d_* = 7.0 μM), showing that the binding of **4** upon HSA was not influenced by HSA bound variable amount of fatty acid. To assign the binding site of **4** on HSA, site-specific binding drugs were used: Ibuprofen (domain IIIA), Digitoxin (domain IIIA), Warfarin (domain IIA), and salicylic acid (domain IIA) [44,45]. By displacement assay using these site-markers, Ibuprofen and Digitoxin led to over 30% decrease in the fluorescence intensity of the **4**-HSA complex and these results clearly indicated that the specific binding of probe **4** at similar binding site of ibuprofen and digitoxin in HSA caused the change on fluorescence enhancement (Figure 4b).

### 3.5. Sensing Mechanism of Probe ***4***

**Figure 5:**
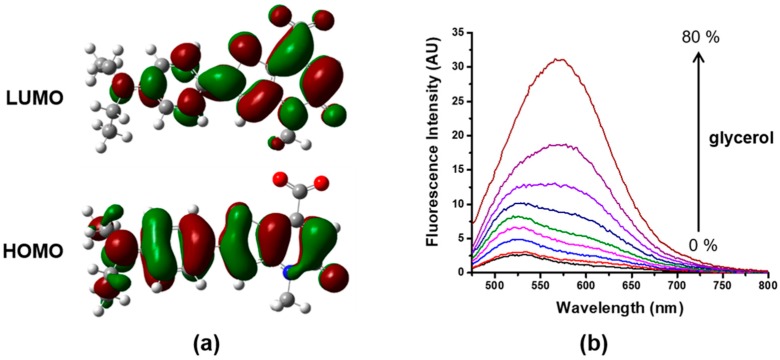
(**a**) Molecular orbitals of fluorophore **4** by DFT calculation, (**b**) Fluorescence spectra of **4** in different fractions of water-glycerol mixtures.

We also carried out DFT calculations to understand the turn-on fluorescence response of probe **4** toward HSA. By performing a DFT/B3LYP/6-311G(d) structural optimization of **4** in the CPCM solvent model (water), we obtained the highest occupied molecular orbital (HOMO) and the lowest unoccupied one (LUMO) of **4**. As shown in Figure 5a, the electron density of HOMO was delocalized along the whole **4** fluorophore, whereas that of LUMO was localized at the thieno[3,2-*b*]pyridine-5(*4H*)-one ring including carboxylate. This HOMO-LUMO transition was assigned as the intramolecular charge transfer (ICT) character of probe **4** [46]. Additionally, we studied the fluorescence behavior of **4** in different water-glycerol fractions and observed fluorescence enhancement by increasing the solvent viscosity (Figure 5b). Several twisted intramolecular charge transfer (TICT)-based fluorogenic probes for various targets, such as biothiol, reactive oxygen species, and HSA, demonstrated the TICT characteristics of the probe through theoretical calculations, showing the charge separation in the HOMO-LUMO transition, and by experimental confirmation of the solvent effect on fluorescence [47,48,49,50,51,52,53]. As in previous reports, these results, including the ICT character and viscosity-dependence of the fluorescence intensity of **4,** showed the typical phenomena of a TICT-based fluorogenic probe. It implied that the inhibition of TICT by a restriction of the rotation of **4,** due to **4** binding to HSA, played an essential role in the turn-on fluorescence response of probe **4** in HSA-sensing.

**Figure 6:**
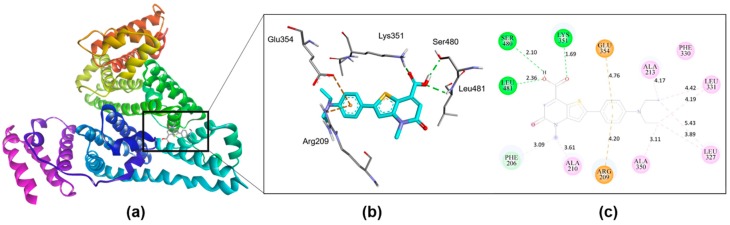
(**a**) The representative binding conformation of 4 in the interface between IIA and IIB of HSA (**b**) Specific residues showing polar interactions between 4 and HSA, (**c**) 2D binding pattern of 4 into HSA binding site.

Molecular docking of the fluorescent probe with HSA was performed to identify the binding mode and intermolecular interactions that would be helpful to reveal the working principle of the fluorescent probe. The binding of **4** with HSA was chosen based on the representative conformation with lowest predicted free energy of binding from the largest cluster. As we expected from our drug displacement experiment, molecular docking results showed that **4** exhibited two possible binding site like Ibuprofen with similar binding mode [45]. **4** was found to bind with HSA on both the primary (subdomain IIIA) and secondary binding site (the interface between subdomain IIA and IIB); the key difference in binding is the reduced intramolecular rotational motion of **4** in the secondary binding site, which is not observed in the primary binding site, as shown in Figure 6 and Figure S23. In the secondary binding site, **4** has energetically favorable binding with HSA via strong intermolecular hydrogen bonds and hydrophobic interactions (Figure 6). The carboxylate groups of probe **4** had hydrogen bond with the sidechain of Ser480 and Lys351 with distance of 2.10Å and 1.69Å respectively along with a hydrogen bond with the backbone of Leu481 with distance of 2.36Å. Additionally, a Pi-Ion interaction were observed between the ‘single ring’ and the sidechain of Arg209 and Glu354. The residue Arg209 was present in a helix that resides in the entrance of the primary drug binding site. Apart from the polar interactions, several (Pi, Alkyl and Mixed Pi/alkyl) hydrophobic interactions were observed with the contact residues Phe206, Ala210, Ala213, Leu327, Phe330, Leu331, and Ala350. On the basis of these results, we propose that the probe **4** shows fluorescence enhancement when it strongly binds at the secondary binding site between subdomain IIA and IIB of HSA.

To confirm the selective recognition of probe **4** toward HSA over BSA which has high structural similarity, comparative docking analysis has been carried out. **4** showed also two possible binding site to BSA, whereas the reduced intramolecular rotational motion of **4** was observed in subdomain IIIA in contrast to HSA (Figure S24). Moreover, probe **4**-HSA complex showed lower predicted free energy of binding (−8.64 kcal/mol) than that of **4**-BSA complex (−5.81 kcal/mol). These results revealed the selective recognition and the different fluorescence-response of **4** toward HSA over BSA. 

### 3.6. Application of Probe ***4*** in Artificial Urine

**Figure 7:**
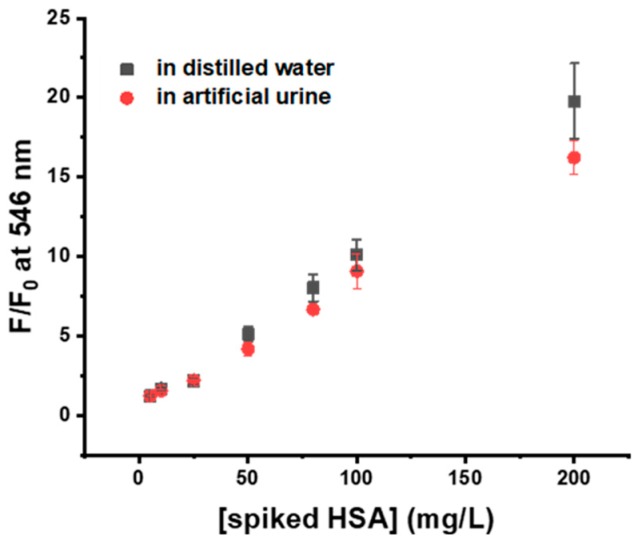
Comparison of the quantification results of the spiked HSA in artificial urine and distilled water, using **4** (5 μM, 10% DMSO) in pH 9 buffer (Tris, 20 mM), λ_ex_ = 455 nm.

To explore the feasibility of using probe **4** for HSA detection in biological samples, we performed a HSA quantitation in artificial urine. An HSA-spiked sample was prepared using artificial urine, varying the range of concentrations of spiked HSA from a fairly low concentration, as the HSA level of normal urine (5–25 mg/L), to a higher concentration simulating microalbuminuria (>30 mg/L). Then, a sample for HSA quantitation was prepared by mixing **4** with a diluted urine sample in pH 9 buffer and the fluorescence intensity was measured. As shown in Figure 7, the fluorescence enhancement induced by the **4**-HSA complex in the artificial urine sample was consistent with that in distilled water, even at very low concentrations. These results demonstrated that probe **4** could be applied to a simple determination of the HSA level in complex biological samples, even for urine containing trace amounts of HSA, and that the quantitation results was highly reliable.

## 4. Conclusions

We have developed a fluorescent probe for sensitive and selective detection of HSA using KF derivatives (fluorescent thieno[3,2-*b*]pyridine-5(*4H*)-one derivatives). By fluorescence-based high-throughput screening of fluorophores against major plasma proteins, we found the potential HSA probe **4** showing a large fluorescence enhancement (160-fold increase) by binding to HSA and characterized its HSA-sensing behavior in terms of sensitivity, selectivity toward HSA, and binding properties. These results demonstrated that **4** exhibited fast response, high sensitivity (LOD 8 nM), and excellent selectivity towards HSA, even in the apparent discrimination between HSA and BSA. In addition, we identified by DFT calculations and molecular docking that the fluorescence turn-on behavior of **4** upon HSA binding was controlled by inhibition of TICT based on strong binding at the interface between IIA and IIB. Moreover, the quantitation of HSA was achieved in an artificial urine sample containing various biological interferences. This probe **4** could be applicable to the determination of trace amounts of HSA in complex biological environments, and thus, to the pre-clinical diagnosis of diverse human diseases.

## Supplementary Materials

The following are available online at https://www.mdpi.com/1424-8220/19/23/5298/s1, Figures S1–S4: ^1^H NMR and ^13^C{^1^H} NMR spectra copies of synthesized compounds **13** and **14**, Table S1, Figures S5–S18: Results of screening of UV-vis absorption of KIOST-Fluors under different buffer condition, Figures S19 and S20: UV-vis spectroscopy study of **4** and **11** in presence of serum albumin (HSA or BSA), Figure S21: Optimization of measurement condition for selective HSA detection using **4**, Figure S22: Determination of the dissociation constant (*K_d_*) of HSA and **4**, Figures S23 and S24: Results of molecular docking studies of **4**.
