# Supplementary material for: Development of Human Serum Albumin Selective Fluorescent Probe Using Thieno[3,2-b]pyridine-5(4H)-one Fluorophore Derivatives
Source: Sensors (Basel). 2019 Dec 1;19(23):5298. doi: 10.3390/s19235298 (PMC6928863; doi:10.3390/s19235298)
Supplement: Supplementary file 1 [file sensors-19-05298-s001.pdf]

sensors

Supplementary Material

Development of Human Serum Albumin Selective Fluorescent Probe Using Thieno[3,2-*b*]pyridine-5(*4H*)-one Fluorophore Derivatives

Suji Lee [1], Dan-Bi Sung [2], Seungyoon Kang [1], Saravanan Parameswaran [1], Jun-Ho Choi [1], Jong Seok Lee [2,3,*] and Min Su Han [1,*]

1 Department of Chemistry, Gwangju Institute of Science and Technology (GIST), 123 Cheomdangwagi-ro, Buk-gu, Gwangju 61005, Korea
2 Marine Natural Products Chemistry Laboratory, Korea Institute of Ocean Science and Technology (KIOST), Busan 49111, Korea
3 Department of Marine Biotechnology, Korea University of Science and Technology, Daejeon 34113, Korea
* Correspondence: jslee@kiost.ac.kr (J.S.L.); happyhan@gist.ac.kr (M.S.H.)

Contents

1. Synthesis of KIOST-Fluors (thieno[3,2-*b*]pyridine-5(*4H*)-one derivatives)
2. ^{1}H NMR and ^{13}C{^{1}H} NMR spectra copies of synthesized compounds
3. Results of screening of UV-vis absorption of KIOST-Fluors under different buffer condition
4. Results of fluorescence-based screening of KIOST-Fluors against major plasma proteins
5. UV-vis spectroscopy study of **4** and **11** in presence of serum albumin (HSA or BSA)
6. Optimization of measurement condition for selective HSA detection using **4**
7. Determination of the dissociation constant (K_d) of HSA and **4**
8. Results of molecular docking studies of **4**

1. Synthesis of KIOST-Fluors (thieno[3,2-*b*]pyridine-5(*4H*)-one derivatives)

1.1. Materials and Instruments

All melting points were recorded on a micro melting point apparatus and are uncorrected. All anhydrous solvents, boronic acids, and other chemical reagents were purchased from Sigma Aldrich, Alfa Aesar and TCI. Starting material, methyl 3-amino-5-bromothiophene-2-carboxylate was purchased from Matrix Scientific Co.. The organic reactions were monitored by thin layer chromatography (TLC) with 0.25-mm pre-coated silica gel plates (Kieselgel 60F254). Flash column chromatography was performed on silica gel (70-230 mesh) using distilled organic solvents. ^{1}H and ^{13}C Spectra were recorded on Varian Unity-Inova 500 MHz and Bruker 600 MHz spectrometer. Chemical shifts are reported as δ (ppm) values relative to chloroform ($CDCl_3$, δ 7.26) and dimethylsulfoxide (DMSO-d_6, δ 2.50) and coupling constant was noted by Hz units. High resolution mass spectroscopy (HRMS) data were obtained by a quadrupole and time-of-flight (TOF) analyzer in Korea Advanced Institute of Science and Technology (KAIST)-Analysis Center for Research Advancement (KARA) in electrospray ionization (ESI) mode.

1.2. General Procedure for the Synthesis of **13, 14**

KF **13** and **14** were synthesized by a BOP ((Benzotriazol-1-yloxy)tris(dimethylamino)phosphonium hexafluorophosphate) promoted regioselective aza-[3+3] cycloaddition of thiophene-3-amine derivatives with α,β-unsaturated carboxylic acids. Thiopehene-3-amine derivatives were synthesized through our previous report [1].

7-acetyl-2-(benzo[d][1,3]dioxol-5-yl)-4-methylthieno[3,2-b]pyridin-5(4H)-one (**13**). To a solution of 5-(Benzo[*d*][1,3]dioxol-5-yl)-N-methylthiophen-3-amine (40 mg, 0.1715 mmol, 1.0 equiv.) and (*E*)-4-

methoxy-4-oxobut-2-enoic acid (23.5 mg, 0.2057 mmol, 1.2 equiv.) in DMF (1.5 mL, 0.1 M), monomethyl fumarate (27.6 mg, 0.2057 mmol, 1.2 equiv.), BOP (92.0 mg, 0.2057 mmol, 1.2 equiv.), and *N*,*N*-diisopropylethylamine (74.0 µL, 0.428 mmol, 2.5 equiv.) were added. The reaction was stirred for 5 min at room temperature. After completion of the reaction, water (2 mL) was added. The crude was extracted with EtOAc (3 mL) three times. The organic layer was dried over Na_2SO_4, filtered, and evaporated in vacuo. The residue was purified by flash column chromatography (Hexane/EtOAc = 1/1, v/v) on silica to afford the product **13** as light-orange solid (20.2 mg, 0.0617 mmol, 36%). m.p: 244-246 °C; ^{1}H NMR (600 MHz, $CDCl_3$) δ 7.23 (dd, *J* = 8.1 Hz and *J* = 1.8 Hz, 1H), 7.22 (d, *J* = 1.8 Hz, 1H), 7.16 (d, *J* = 1.2 Hz, 1H), 7.10 (d, *J* = 10.2 Hz, 1H), 6.86 (d, *J* = 8.4 Hz, 1H), 6.03 (s, 2H), 3.77 (s, 3H), 2.65 (s. 3H); ^{13}C{^{1}H} NMR (150 MHz, $CDCl_3$) δ 197.8, 163.0, 152.6, 148.9, 148.6, 146.0, 139.0, 127.7, 120.6, 118.7, 113.2, 110.0, 109.1, 106.7, 101.8, 32.3, 26.3; HRMS (ESI): m/z calcd for $C_{17}H_{13}NNaO_4S$ [M+Na]$^+$ 350.0463, found 350.0449.

*7-acetyl-4-methyl-2-(3,4,5-trimethoxyphenyl)thieno[3,2-b]pyridin-5(4H)-one (**14**).* To a solution of N-Methyl-5-(3,4,5-trimethoxyphenyl)thiophen-3-amine (39.1 mg, 0.140 mmol, 1.0 equiv.) and (*E*)-4-methoxy-4-oxobut-2-enoic acid (19 mg, 0.168 mmol, 1.2 equiv.) in DMF (1.5 mL, 0.1 M), monomethyl fumarate (22.5 mg, 0.168 mmol, 1.2 equiv.), BOP (75.0 mg, 0.168 mmol, 1.2 equiv.), and *N*,*N*-diisopropylethylamine (60.0 µL, 0.35 mmol, 1.2 equiv.) were added. The reaction was stirred for 5 min at room temperature. After completion of the reaction, water (2 mL) was added. The crude was extracted with EtOAc (3 mL) three times. The organic layer was dried over Na_2SO_4, filtered, and evaporated in vacuo. The residue was purified by flash column chromatography (Hexane/EtOAc = 1/1, v/v) on silica to obtain the product **14** as yellow solid (13.2 mg, 0.0353 mmol, 26%). m.p: 206-208 °C; ^{1}H NMR (600 MHz, $CDCl_3$) δ 7.15 (s, 1H), 7.11 (s, 1H), 6.89 (s, 2H), 3.94 (s, 6H), 3.89 (s, 3H), 3.80 (s, 3H), 2.65 (s, 3H); ^{13}C{^{1}H} NMR (150 MHz, $CDCl_3$) δ 197.9, 162.9, 153.9, 152.8, 145.8, 139.5, 139.0, 129.1, 119.0, 113.5, 110.5, 103.8, 61.2, 56.5, 32.4, 26.3; HRMS (ESI): m/z calcd for $C_{19}H_{19}NNaO_5S$ [M+Na]$^+$ 396.0882, found 396.0874.

2. ^{1}H NMR and ^{13}C{^{1}H} NMR spectra copies of synthesized compounds 13 and 14

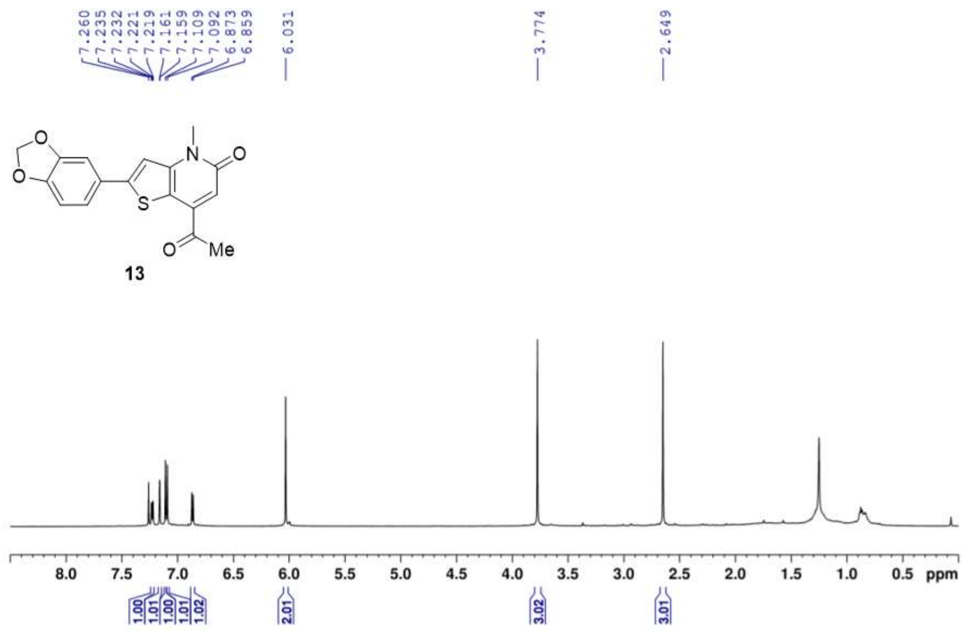

Figure S1. ^{1}H NMR spectra of methyl 7-acetyl-2-(benzo[*d*][1,3]dioxol-5-yl)-4-methylthieno[3,2-*b*]pyridin-5(4*H*)-one (**13**) in $CDCl_3$.

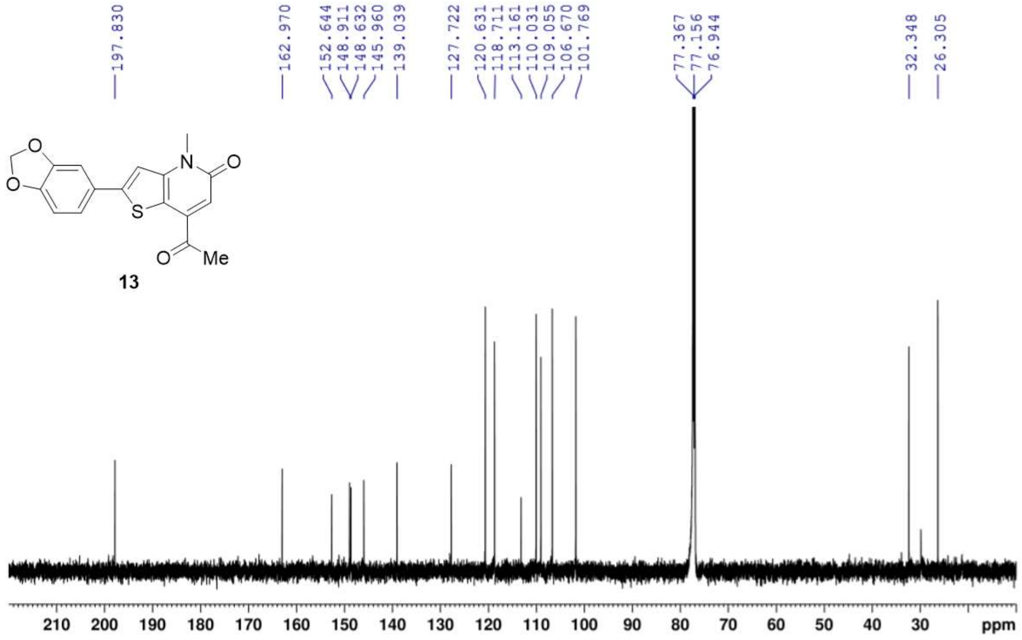

Figure S2. ^{13}C NMR spectra of 7-acetyl-2-(benzo[*d*][1,3]dioxol-5-yl)-4-methylthieno[3,2-*b*]pyridin-5(4*H*)-one (**13**) in $CDCl_3$.

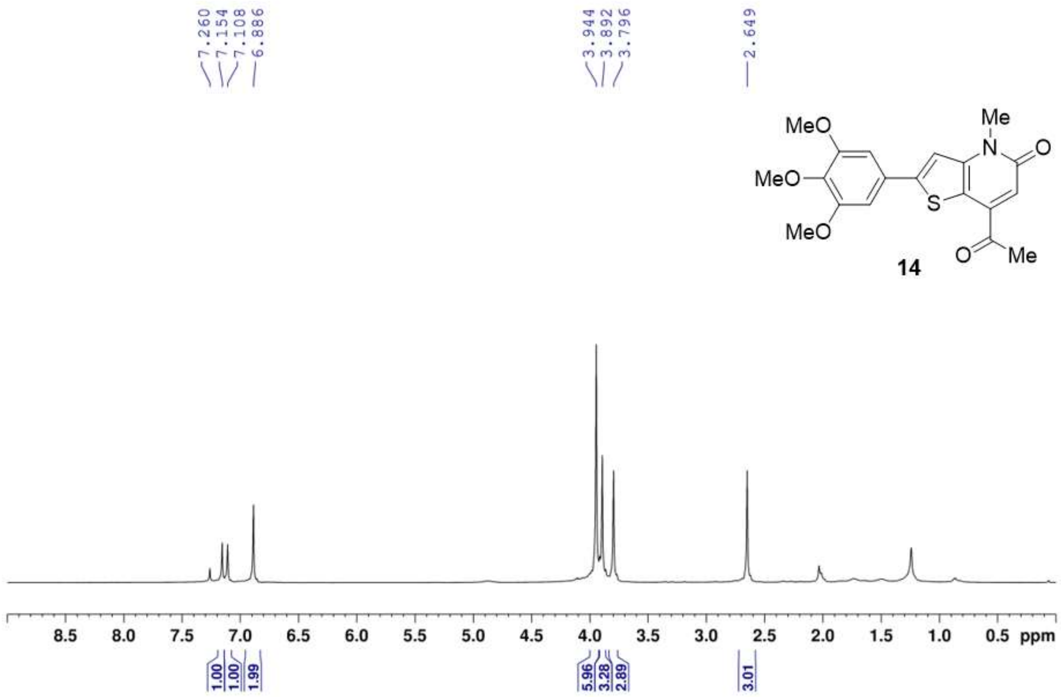

Figure S3. ^{1}H NMR spectra of 7-acetyl-4-methyl-2-(3,4,5-trimethoxyphenyl)thieno[3,2-*b*]pyridin-5(4*H*)-one (**14**) in $CDCl_3$.

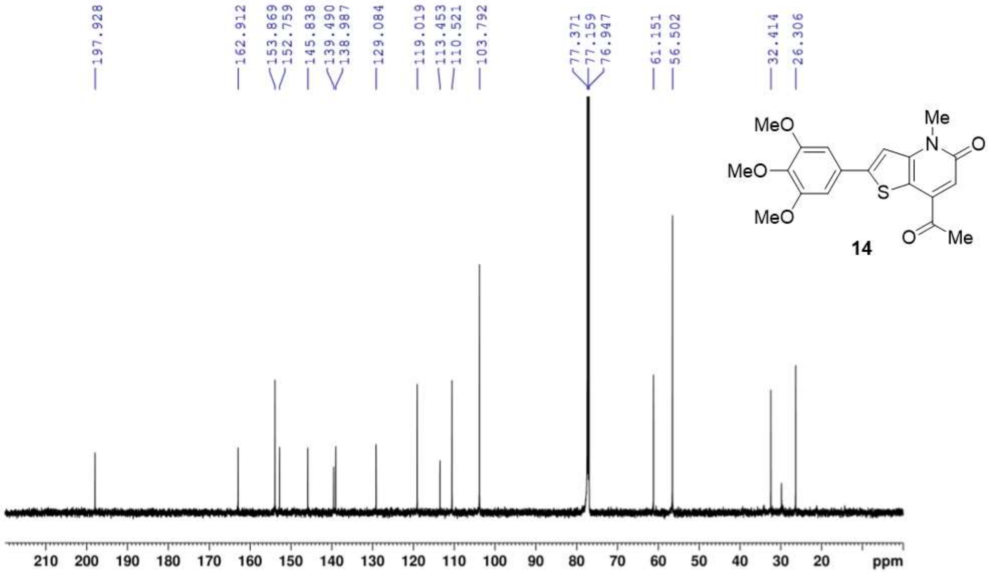

Figure S4. ^{13}C NMR spectra of 7-acetyl-4-methyl-2-(3,4,5-trimethoxyphenyl)thieno[3,2-*b*]pyridin-5(4*H*)-one (**14**) in $CDCl_3$.

3. Results of Screening of UV-Vis Absorption of KIOST-Fluors under Different Buffer Condition

Table S1. Absorption wavelength of KIOST-Fluors (1 – 14) (5 μM) under different buffer condition (pH 5 acetate, pH 7 Tris, pH 9 Tris, 20 mM) in 10% DMSO.

	**λ_{abs} a)**		
	**pH 5**	**pH 7**	**pH 9**
**1**	400 nm	400 nm	400 nm
**2**	400 nm	400 nm	400 nm
**3**	405 nm	405 nm	405 nm
**4**	420 nm	420 nm	420 nm
**5**	450 nm	450 nm	450 nm
**6**	390 nm	390 nm	390 nm
**7**	385 nm	385 nm	385 nm
**8**	400 nm	400 nm	400 nm
**9**	410 nm	410 nm	410 nm
**10**	420 nm	420 nm	420 nm
**11**	420 nm	430 nm	430 nm
**12**	375 nm	375 nm	375 nm
**13**	410 nm	410 nm	410 nm
**14**	405 nm	405 nm	405 nm

a) The absorption wavelength having maximum optical density.

4. Results of Fluorescence-Based High-Throughput Screening of KIOST-Fluors against Major Plasma Proteins

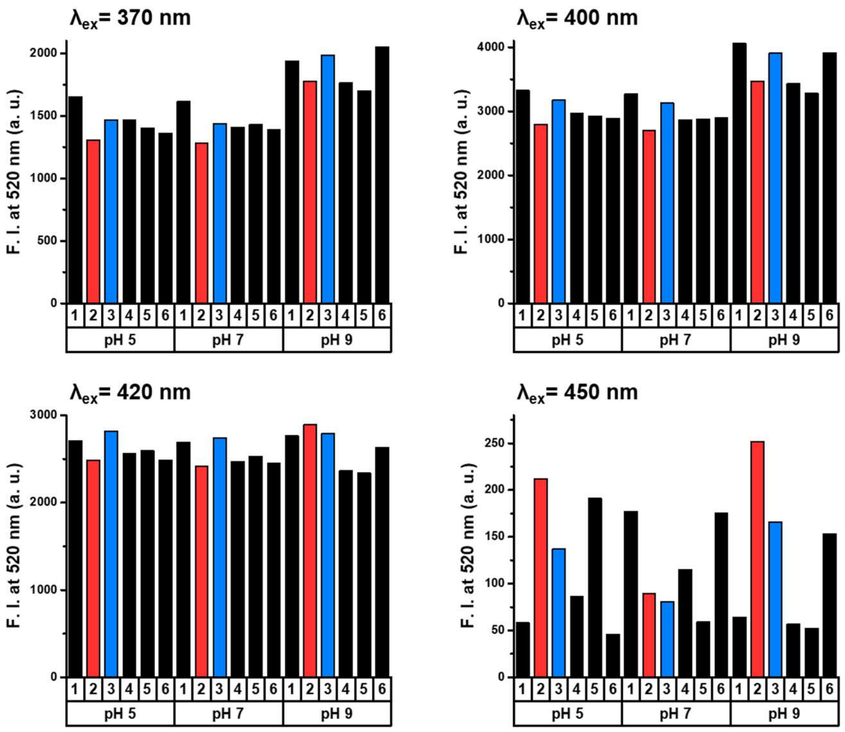

Figure S5. Fluorescence intensity of fluorophore **1** (5 μM) in absence and presence of major plasma proteins (10 μM) under different buffer condition (pH 5 acetate, pH 7 Tris, pH 9 Tris; 20 mM) in 10% DMSO, 1: blank, 2: HSA, 3: BSA, 4: γ-globulin, 5: fibrinogen, 6: transferrin.

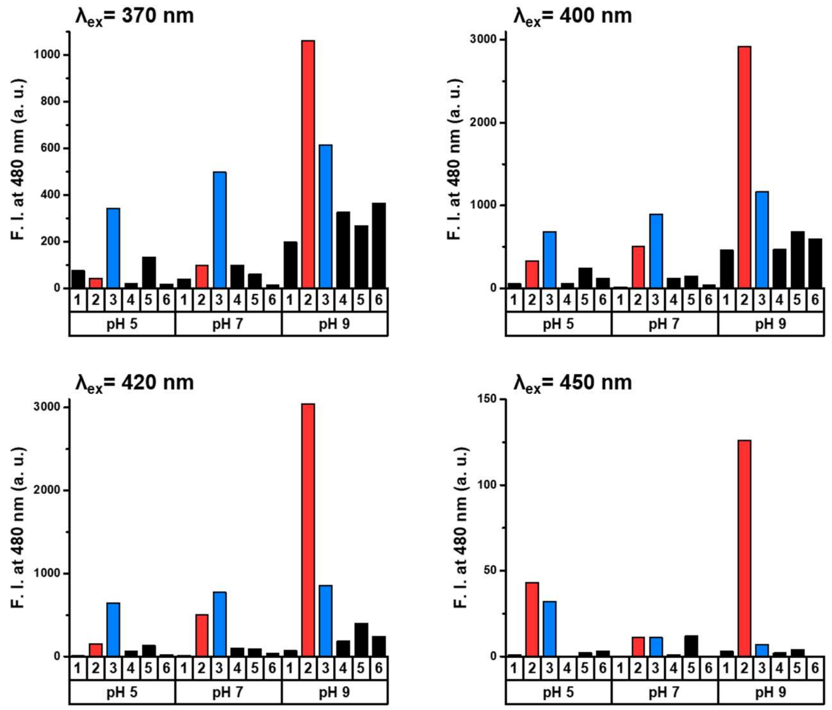

Figure S6. Fluorescence intensity of fluorophore **2** (5 μM) in absence and presence of major plasma proteins (10 μM) under different buffer condition (pH 5 acetate, pH 7 Tris, pH 9 Tris; 20 mM) in 10% DMSO, 1: blank, 2: HSA, 3: BSA, 4: γ-globulin, 5: fibrinogen, 6: transferrin.

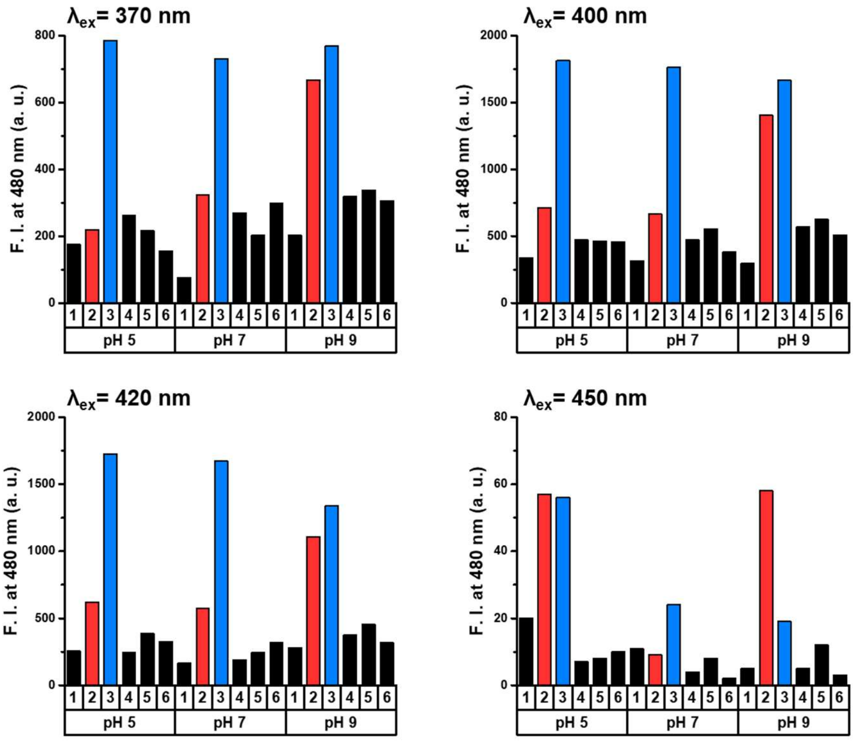

Figure S7. Fluorescence intensity of fluorophore **3** (5 μM) in absence and presence of major plasma proteins (10 μM) under different buffer condition (pH 5 acetate, pH 7 Tris, pH 9 Tris; 20 mM) in 10% DMSO, 1: blank, 2: HSA, 3: BSA, 4: γ-globulin, 5: fibrinogen, 6: transferrin.

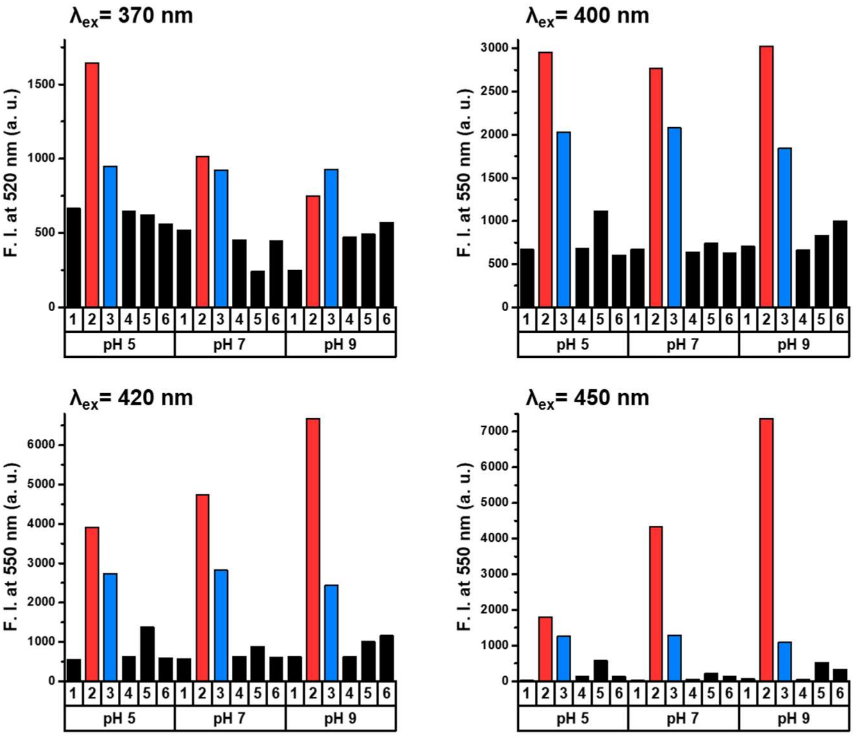

Figure S8. Fluorescence intensity of fluorophore **4** (5 μM) in absence and presence of major plasma proteins (10 μM) under different buffer condition (pH 5 acetate, pH 7 Tris, pH 9 Tris; 20 mM) in 10% DMSO, 1: blank, 2: HSA, 3: BSA, 4: γ-globulin, 5: fibrinogen, 6: transferrin.

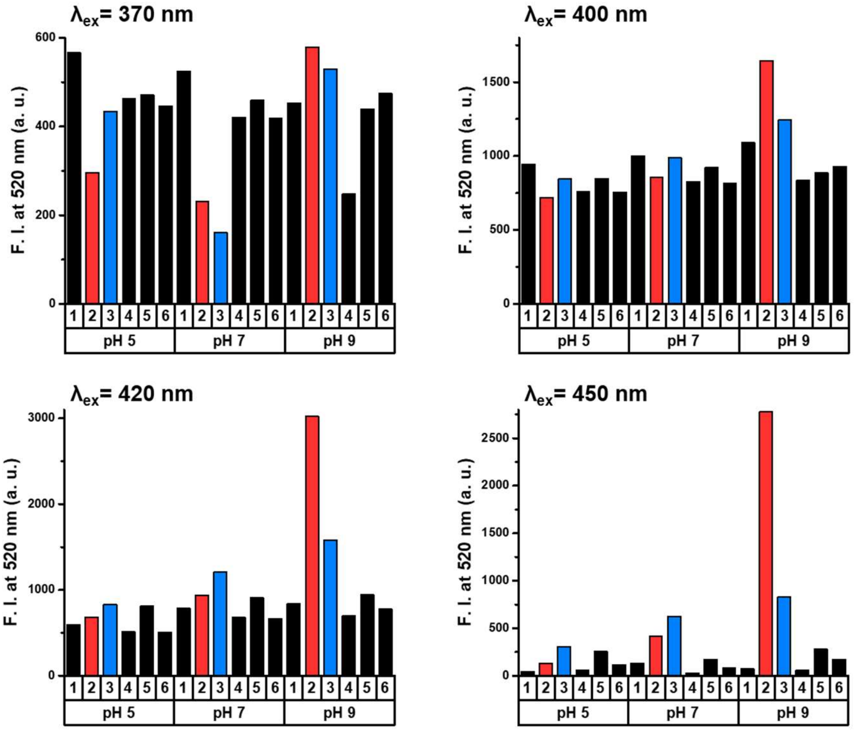

Figure S9. Fluorescence intensity of fluorophore **5** (5 μM) in absence and presence of major plasma proteins (10 μM) under different buffer condition (pH 5 acetate, pH 7 Tris, pH 9 Tris; 20 mM) in 10% DMSO, 1: blank, 2: HSA, 3: BSA, 4: γ-globulin, 5: fibrinogen, 6: transferrin.

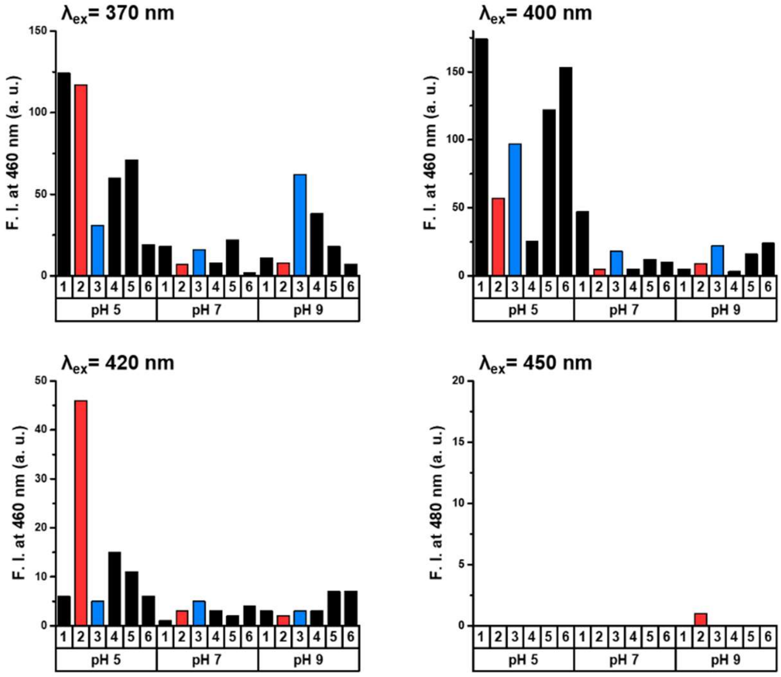

Figure S10. Fluorescence intensity of fluorophore **6** (5 μM) in absence and presence of major plasma proteins (10 μM) under different buffer condition (pH 5 acetate, pH 7 Tris, pH 9 Tris; 20 mM) in 10% DMSO, 1: blank, 2: HSA, 3: BSA, 4: γ-globulin, 5: fibrinogen, 6: transferrin.

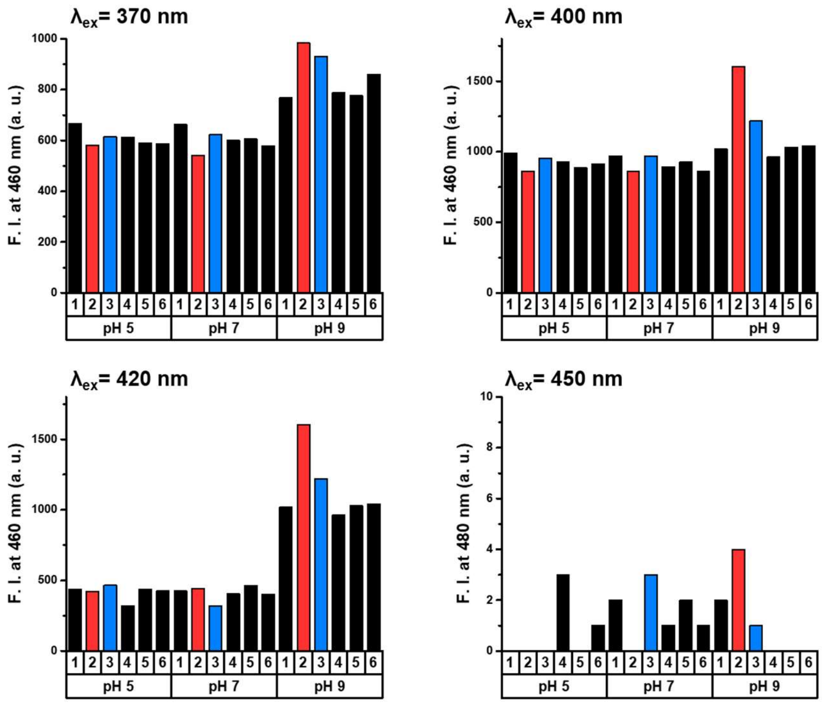

Figure S11. Fluorescence intensity of fluorophore **7** (5 μM) in absence and presence of major plasma proteins (10 μM) under different buffer condition (pH 5 acetate, pH 7 Tris, pH 9 Tris; 20 mM) in 10% DMSO, 1: blank, 2: HSA, 3: BSA, 4: γ-globulin, 5: fibrinogen, 6: transferrin.

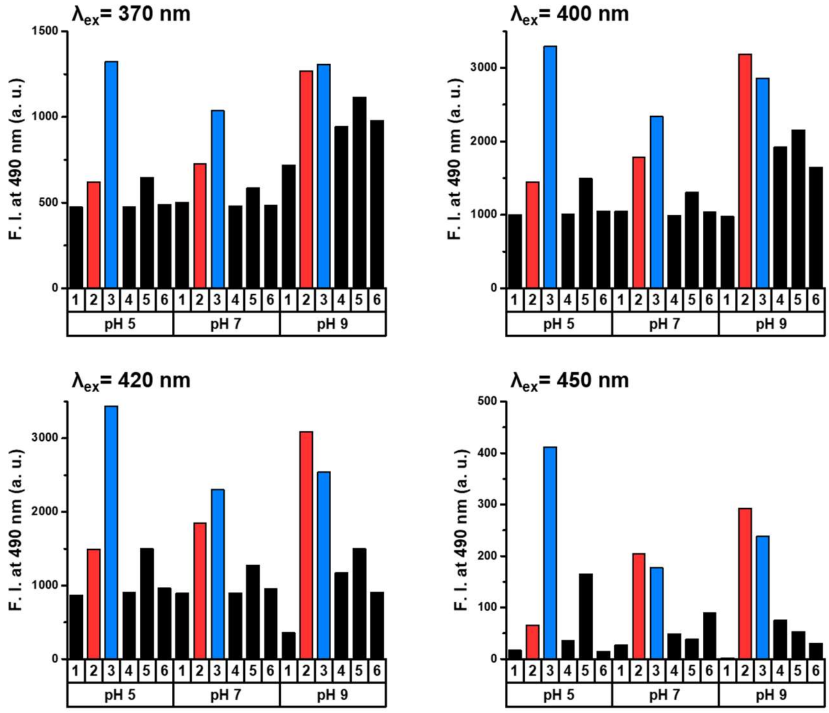

Figure S12. Fluorescence intensity of fluorophore **8** (5 μM) in absence and presence of major plasma proteins (10 μM) under different buffer condition (pH 5 acetate, pH 7 Tris, pH 9 Tris; 20 mM) in 10% DMSO, 1: blank, 2: HSA, 3: BSA, 4: γ-globulin, 5: fibrinogen, 6: transferrin.

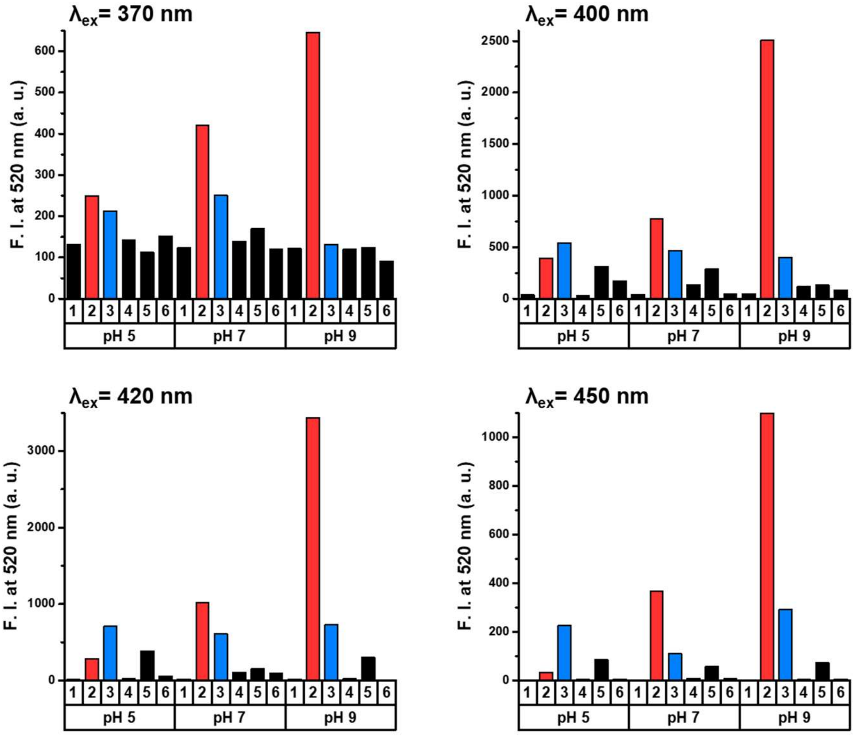

Figure S13. Fluorescence intensity of fluorophore **9** (5 μM) in absence and presence of major plasma proteins (10 μM) under different buffer condition (pH 5 acetate, pH 7 Tris, pH 9 Tris; 20 mM) in 10% DMSO, 1: blank, 2: HSA, 3: BSA, 4: γ-globulin, 5: fibrinogen, 6: transferrin.

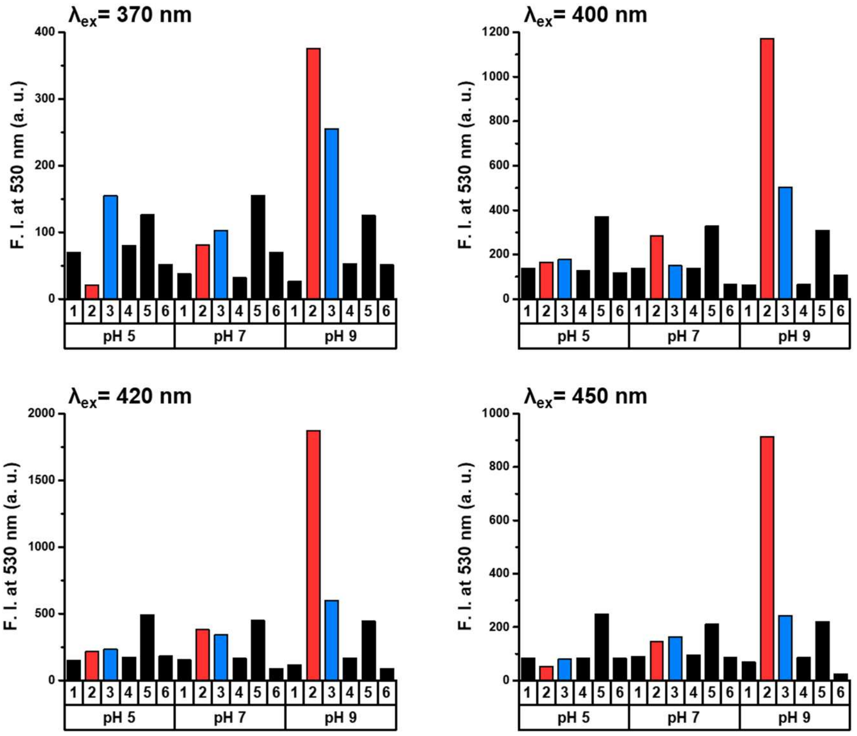

Figure S14. Fluorescence intensity of fluorophore **10** (5 μM) in absence and presence of major plasma proteins (10 μM) under different buffer condition (pH 5 acetate, pH 7 Tris, pH 9 Tris; 20 mM) in 10% DMSO, 1: blank, 2: HSA, 3: BSA, 4: γ-globulin, 5: fibrinogen, 6: transferrin.

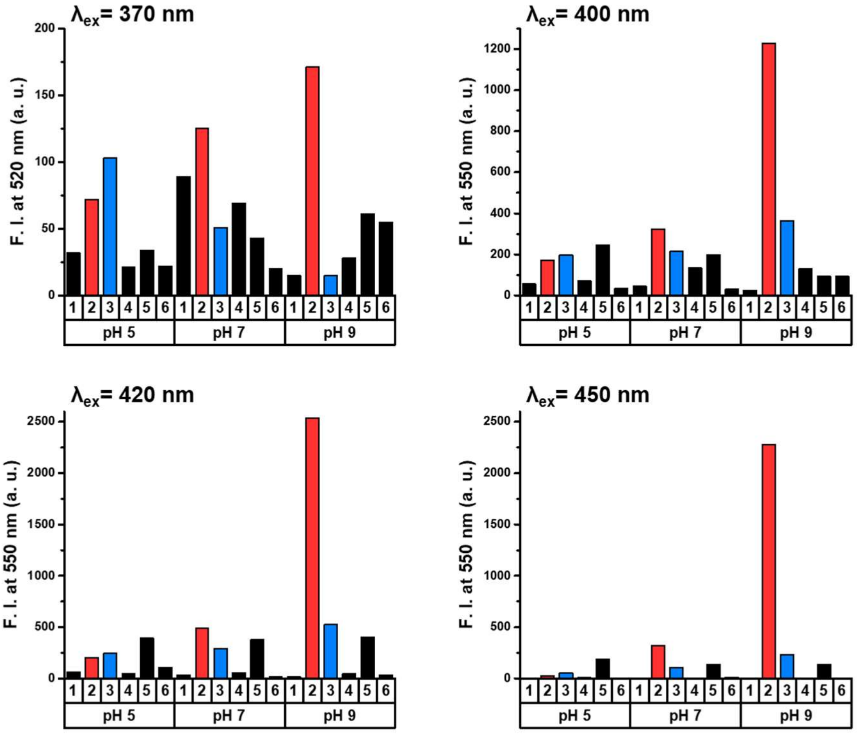

Figure S15. Fluorescence intensity of fluorophore **11** (5 μM) in absence and presence of major plasma proteins (10 μM) under different buffer condition (pH 5 acetate, pH 7 Tris, pH 9 Tris; 20 mM) in 10% DMSO, 1: blank, 2: HSA, 3: BSA, 4: γ-globulin, 5: fibrinogen, 6: transferrin.

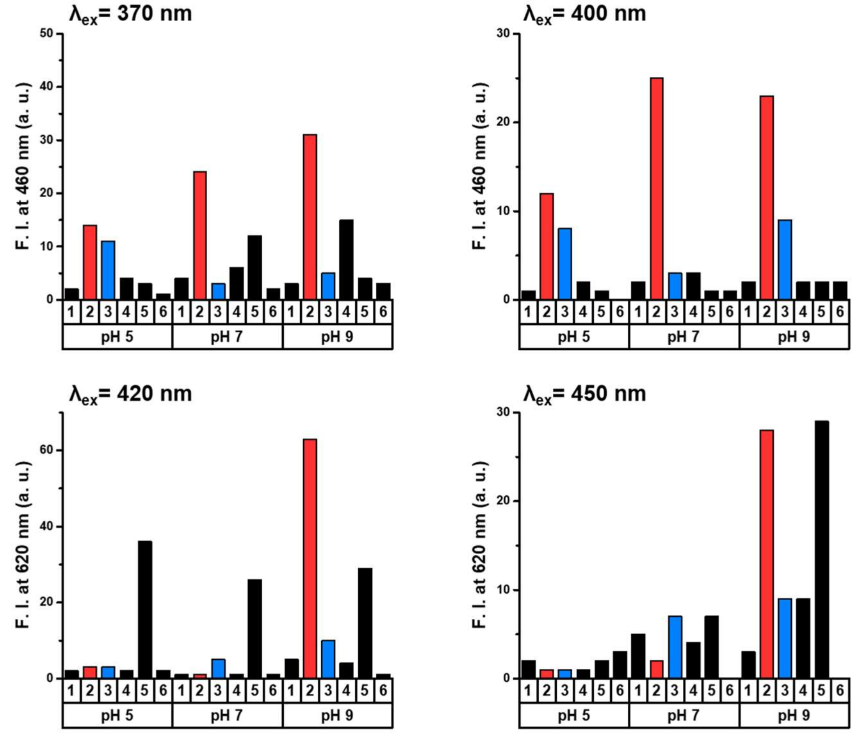

Figure S16. Fluorescence intensity of fluorophore **12** (5 μM) in absence and presence of major plasma proteins (10 μM) under different buffer condition (pH 5 acetate, pH 7 Tris, pH 9 Tris; 20 mM) in 10% DMSO, 1: blank, 2: HSA, 3: BSA, 4: γ-globulin, 5: fibrinogen, 6: transferrin.

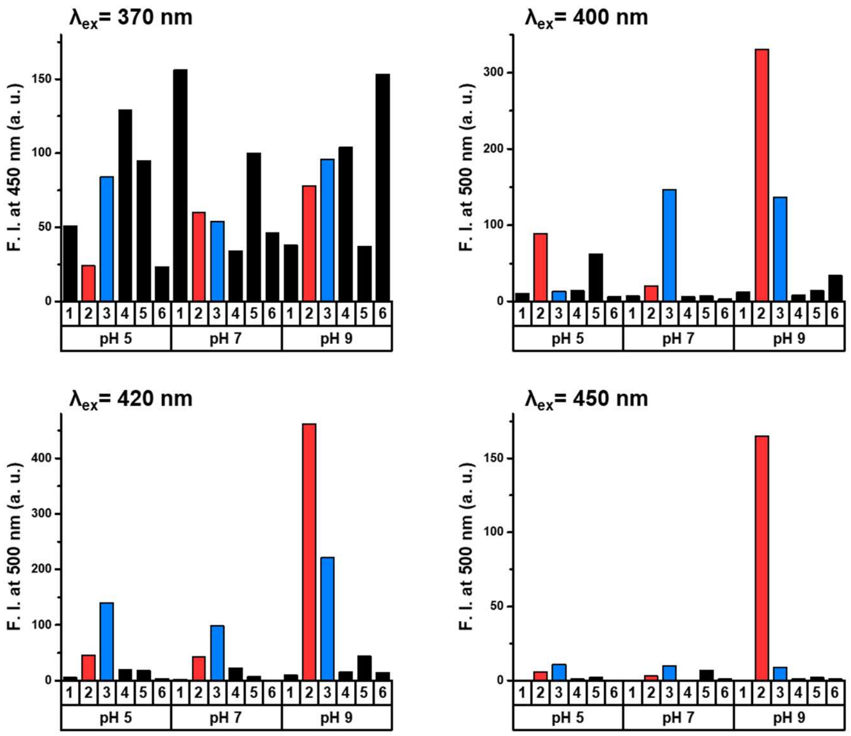

Figure S17. Fluorescence intensity of fluorophore **13** (5 μM) in absence and presence of major plasma proteins (10 μM) under different buffer condition (pH 5 acetate, pH 7 Tris, pH 9 Tris; 20 mM) in 10% DMSO, 1: blank, 2: HSA, 3: BSA, 4: γ-globulin, 5: fibrinogen, 6: transferrin.

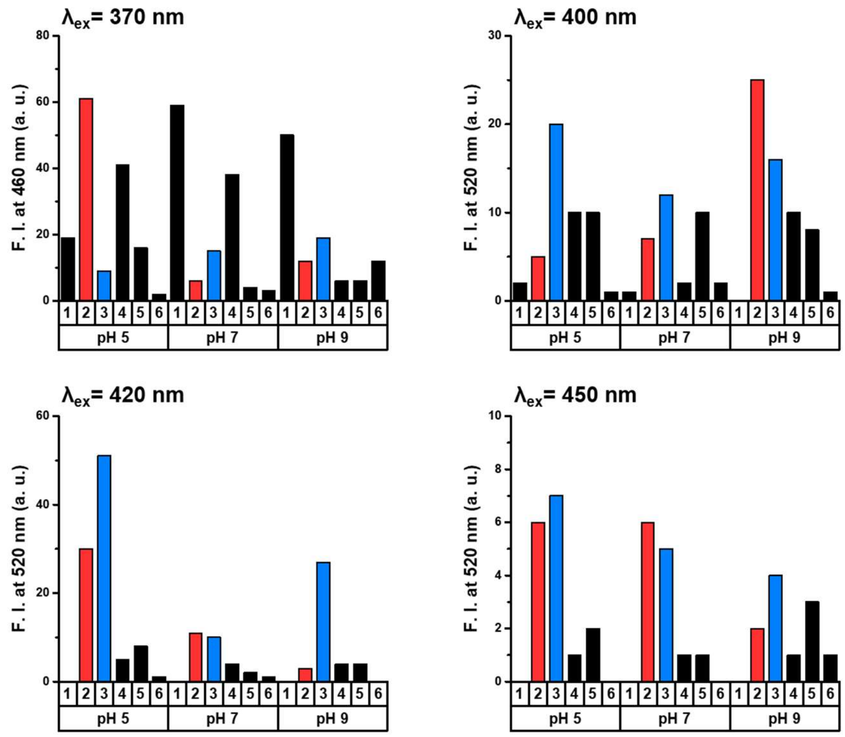

Figure S18. Fluorescence intensity of fluorophore **14** (5 μM) in absence and presence of major plasma proteins (10 μM) under different buffer condition (pH 5 acetate, pH 7 Tris, pH 9 Tris; 20 mM) in 10% DMSO, 1: blank, 2: HSA, 3: BSA, 4: γ-globulin, 5: fibrinogen, 6: transferrin.

5. UV-Vis Spectroscopy Study of 4 and 11 in Presence of Serum Albumin (HSA or BSA)

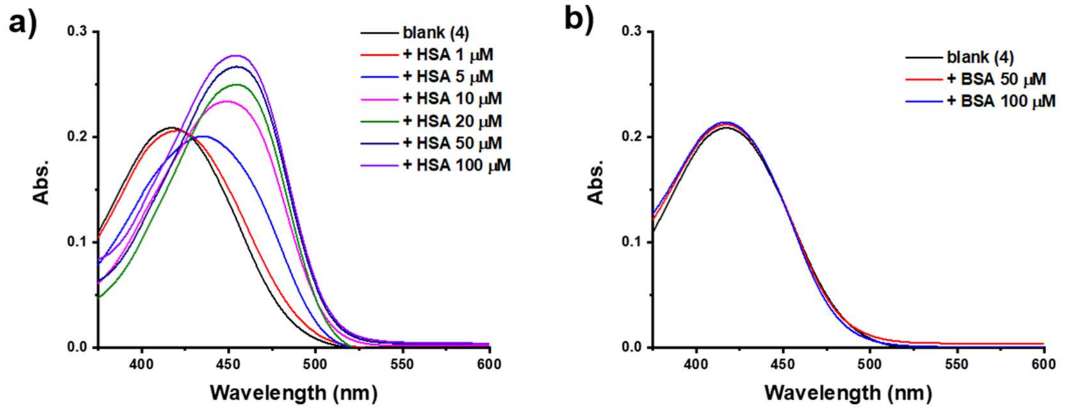

Figure S19. UV-vis spectra of **4** (5 μM, 10% DMSO) in pH 9 buffer (Tris, 20 mM), (**a**) with various concentration of HSA, (**b**) with high concentration of BSA.

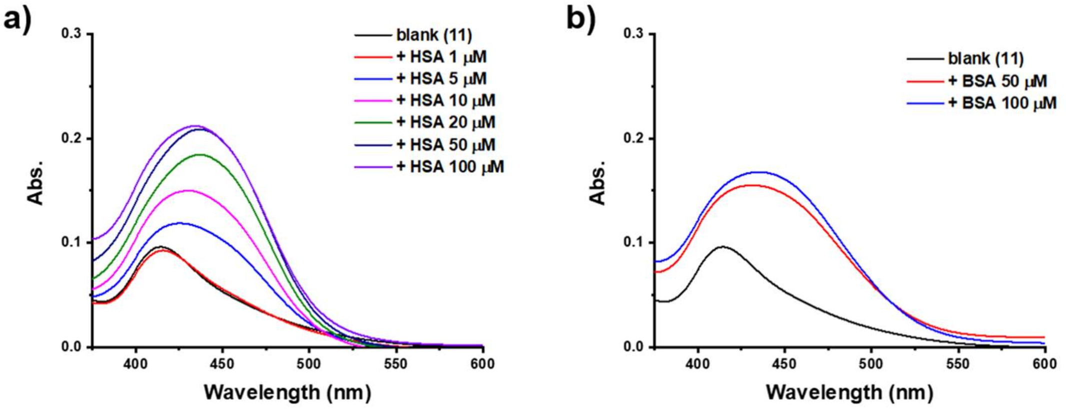

Figure S20. UV-vis spectra of **11** (5 μM, 10% DMSO) in pH 9 buffer (Tris, 20 mM), (**a**) with various concentration of HSA, (**b**) with high concentration of BSA.

6. Optimization of Measurement Condition for Selective HSA Detection using 4

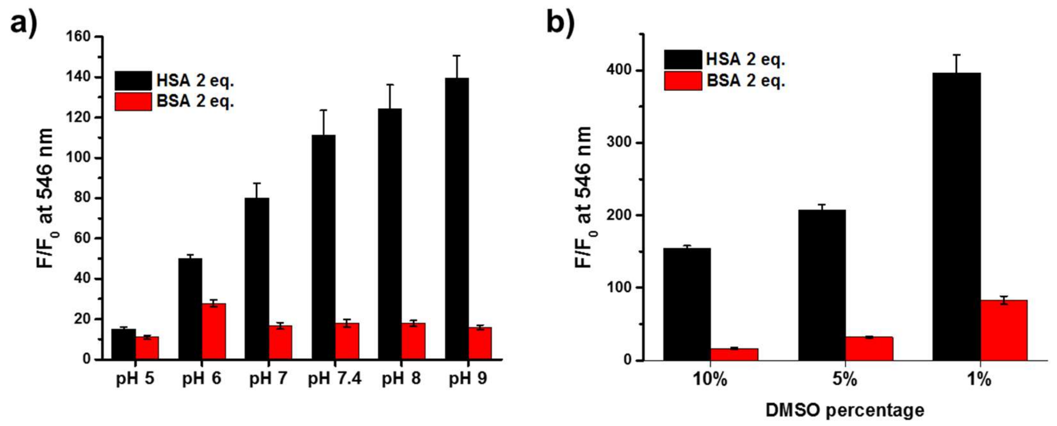

Figure S21. Fluorescence enhancement of fluorophore **4** (5 μM) in presence of HSA or BSA (10 μM), (**a**) in different pH condition (pH 5, 6 acetate, pH 7, 7.4, 8, 9 Tris; 20 mM) in 10% DMSO, (**b**) in different ratio of DMSO solvent in pH 9 (Tris, 20 mM), λ_{ex} = 455 nm.

7. Determination of the Dissociation Constant (K_d) of HSA and 4

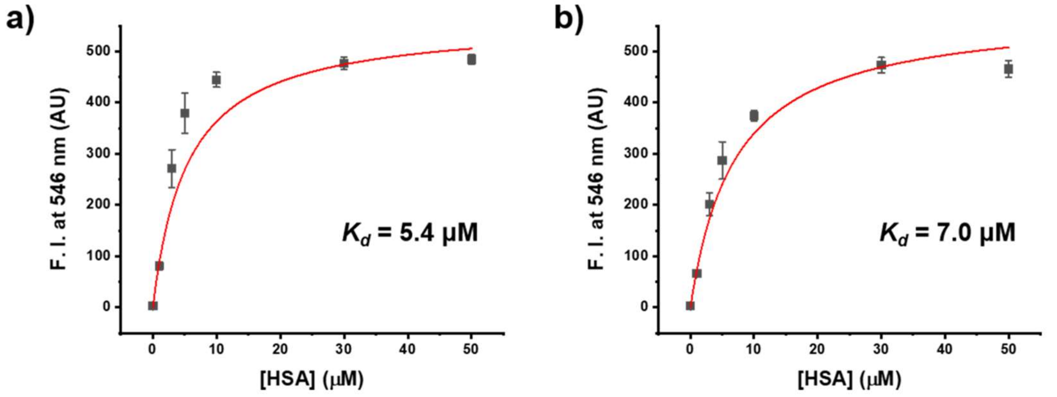

Figure S22. One-site specific binding curve showing the change in fluorescence intensity at 546 nm of probe **4** (5 μM, 10% DMSO) in pH 9 buffer (Tris, 20 mM) with (**a**) native HSA, (**b**) delipidated HSA.

8. Results of Molecular Docking Studies of 4

8.1. Docking Conformation of 4-HSA Complex

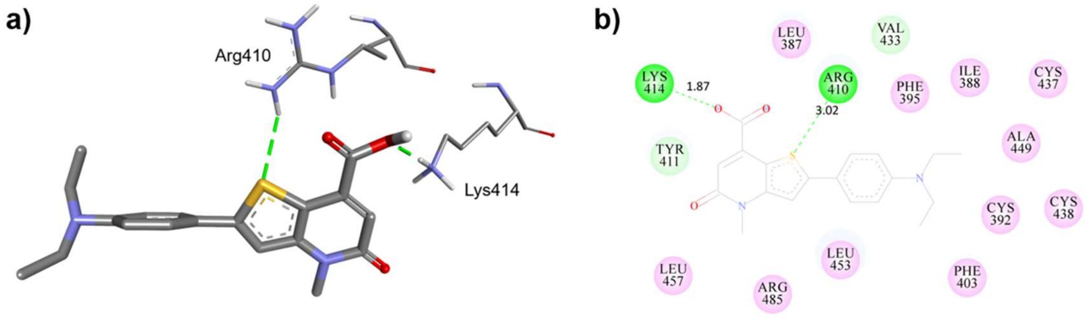

Figure S23. (**a**) Hydrogen bonding interactions between **4** and HSA, (**b**) 2D binding pattern of **4** into HSA binding site IIIA.

8.2. Docking Conformation of 4-BSA Complex

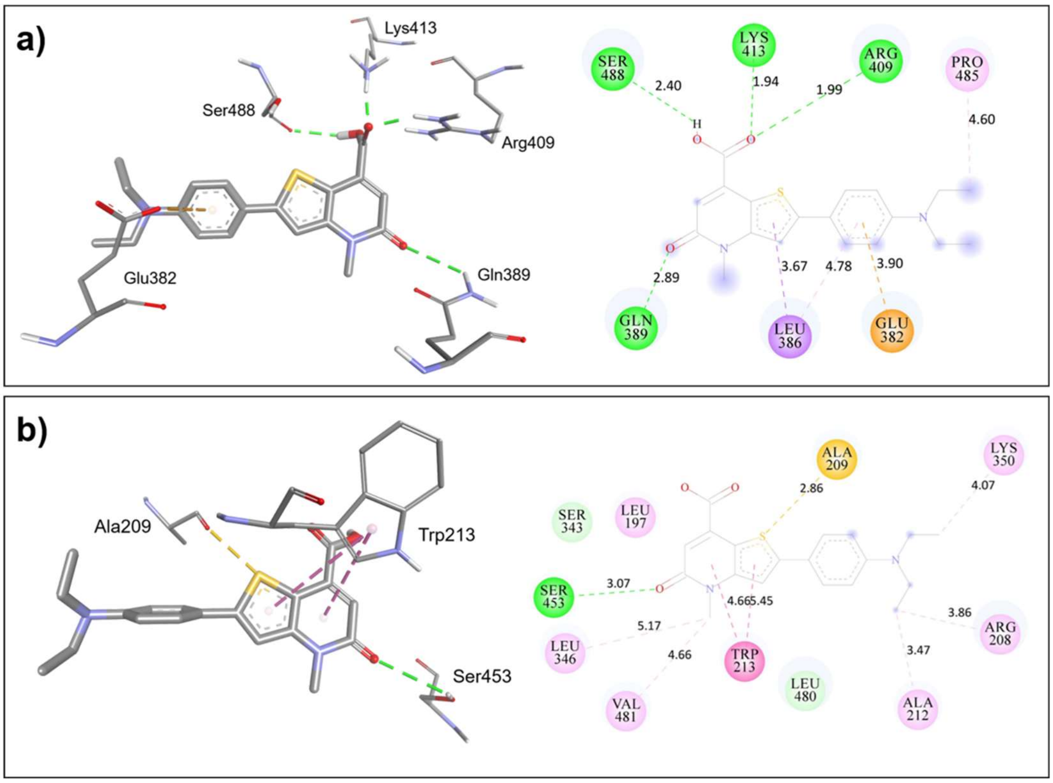

Figure S24. The docked images of **4**-BSA complexes, (**a**) at the primary (subdomain IIIA) and (**b**) at the secondary (interface between IIA and IIB) binding sites, specific residues showing polar interactions (left) and 2D binding pattern of **4** into each binding site (right).

Reference

[1] Sung, D.-B.; Mun, B.; Park, S.; Lee, H.-S.; Lee, J.; Lee, Y.-J.; Shin, H.J.; Lee, J.S. Synthesis, Molecular Engineering, and Photophysical Properties of Fluorescent Thieno[3,2-*b*]pyridine-5(*4H*)-ones. *J. Org. Chem.* **2019**, *84*, 379.
